# Simultaneous Pretreatment of Aspirin and Omega-3 Fatty Acid Attenuates Nuclear Factor-κB Activation in a Murine Model with Ventilator-Induced Lung Injury

**DOI:** 10.3390/nu13072258

**Published:** 2021-06-30

**Authors:** Won-Gun Kwack, Yoon-Je Lee, Eun-Young Eo, Jin-Haeng Chung, Jae-Ho Lee, Young-Jae Cho

**Affiliations:** 1 Division of Pulmonary, Allergy and Critical Care Medicine, Department of Internal Medicine, Kyung Hee University Hospital, Seoul 02447, Korea; wongunnim@naver.com; 2 Division of Pulmonary and Critical Care Medicine, Department of Internal Medicine, Seoul National University College of Medicine, Seoul National University Bundang Hospital, Seongnam 13620, Korea; r1950@snubh.org (Y.-J.L.); r0713@snubh.org (E.-Y.E.); Jhlee7@snubh.org (J.-H.L.); 3 Department of Pathology, Seoul National University College of Medicine, Seoul National University Bundang Hospital, Seongnam 13620, Korea; jhchung@snubh.org

**Keywords:** aspirin, nuclear factor-κB, tumor necrosis factor-α, omega-3 fatty acid, ventilator-induced lung injury

## Abstract

Ventilator-induced lung injury (VILI) is an important critical care complication. Nuclear factor-κB (NF-κB) activation, a critical signaling event in the inflammatory response, has been implicated in the tracking of the lung injury. The present study aimed to determine the effect of simultaneous pretreatment with enteral aspirin and omega-3 fatty acid on lung injury in a murine VILI model. We compared the lung inflammation after the sequential administration of lipopolysaccharides and mechanical ventilation between the pretreated simultaneous enteral aspirin and omega-3 fatty acid group and the non-pretreatment group, by quantifying NF-κB activation using an in vivo imaging system to detect bioluminescence signals. The pretreated group with enteral aspirin and omega-3 fatty acid exhibited a smaller elevation of bioluminescence signals than the non-pretreated group (*p* = 0.039). Compared to the non-pretreated group, the pretreatment group with simultaneous enteral aspirin and omega-3 fatty acid showed reduced expression of the pro-inflammatory cytokine, tumor necrosis factor-α, in bronchoalveolar lavage fluid (*p* = 0.038). Histopathological lung injury scores were also lower in the pretreatment groups compared to the only injury group. Simultaneous pretreatment with enteral administration of aspirin and omega-3 fatty acid could be a prevention method for VILI in patients with impending mechanical ventilation therapy.

## 1. Introduction

Since the introduction of mechanical ventilation (MV) in the 1960s, it has been widely adopted in clinical practices, and inevitably ventilator-induced lung injury (VILI) has become an important topic for study in critical care. Physical forces exerted during MV may induce cell injury or activate cell signaling pathways that subsequently lead to the release of various intra- and inter-cellular mediators, resulting in direct or indirect lung injury. Excessive stretching of the lungs, which induces plasma membrane disruption and cytoskeletal responses, can trigger acute lung inflammation via the activation of nuclear factor kappa-light-chain-enhancer of activated B cells (NF-κB) [1], a heterodimer consisting of subunits, and plays an important role in innate immunity and inflammation [2]. Several researchers have shown that blockades to the NF-κB pathway can attenuate inflammatory responses to MV and decrease the incidence and severity of lung injury [3,4]. 

Omega-3 fatty acids such as docosahexaenoic acid and eicosapentaenoic acid have been suggested as beneficial agents for the reduction in inflammatory processes by inhibiting the arachidonic acid metabolism and altering the expression of inflammatory genes which effect transcription factor activation [5]. Enriched omega-3 fatty acid formula supplement has been associated with reduced length of intensive care unit stay and mortality in mechanically ventilated patients [6,7]. Patients at risk of developing acute lung injury (ALI) and with established ALI had low levels of omega-3 compared to normal (25% and 6% of normal, respectively) [8]. 

Aspirin also represents a positive effect to elicit reduction in the ALI by attenuating inflammatory mediators and increasing restitution of pulmonary barrier function and resolution of injury [9]. In a large cohort study, prehospital aspirin usage was found to be associated with decreased acute respiratory distress syndrome (ARDS) risk. In fact, the metabolism of omega-3 fatty acid can also be affected by aspirin. However, the combination effects of these two drugs on VILI have yet to be fully clarified, especially their association with the NF-κB inflammatory pathway. Therefore, we performed this study to explore the ability of pretreated simultaneous enteral aspirin and omega-3 fatty acid to attenuate lung inflammation in the murine VILI model.

## 2. Materials and Methods

### 2.1. Animals and Study Design

Female C57BL/6 mice (Orient Bio, Seongnam, Korea) aged 11 weeks and weighing 19–21 g, were used in this study. All mice were kept in specific pathogen-free areas with access restricted to only animal care staff. Mice were housed in groups with controlled temperature, humidity, and light. Food and water were available ad libitum. After random selection by a veterinarian who was blinded to the information about the assigned group, the mice were allocated to each experimental group (control, injury only, and pretreatment groups). The control group had sham tracheostomy and transfection of the NF-κB-luciferase reporter (Luc), but neither lung injury nor pretreatment. The injury-only group had MV injury without pretreatment. The pretreatment groups had intravenous resolvin D1 (RvD1) injection or simultaneous enteral aspirin, and omega-3 fatty acid before the lung injury. We compared the changes in NF-κB signal activity 4 h after the lung injury among injury only, RvD1 pretreatment, and pretreated simultaneous enteral aspirin and omega-3 fatty acid groups using an in vivo imaging system to detect the bioluminescence signal. We also compared the total protein and inflammatory cytokines in the bronchoalveolar lavage fluid (BALF) and the histopathologic findings among the groups. All experiments were conducted in accordance with the institutional guidelines and approved by the Seoul National University Bundang Hospital and the Institutional Animal Care and Use Committee (55-2014-048). 

### 2.2. Ventilator-Induced Lung Injury Model 

For induction of anesthesia, mixtures of 12 mg/kg tiletamine-zolazepam (Zoletil; Virbac Laboratories, Carros, France) and 4 mg/kg xylazine (Rompun; Bayer Korea, Ansan, Korea) were diluted 1:10 in normal saline solution, upon which 200 µL of this mixture was administered intraperitoneally. After induction of anesthesia, the mice were placed in a supine position. To ensure a stable body temperature, the mice were placed on a temperature-controlled heated table. A tracheostomy was performed, and a 22-gauge catheter was inserted into the trachea. Thereafter, the mice were immediately attached to a rodent mechanical ventilator (Harvard Apparatus, Boston, MA, USA) with a fractional inspired oxygen concentration of 0.5, tidal volume of 30 mL/kg, and respiratory rate of 120 breaths per min, with zero positive end-expiratory pressure for 4 h. During the period of mechanical ventilation, anesthesia was maintained with isoflurane. To investigate the time-point at which maximal injury was caused, the NF-κB bioluminescence images were checked at 0, 3, 4, 5, and 6 h after completion of the ALI process. Time zero indicated the completion of the 4 h MV application. 

### 2.3. Anti-Inflammatory Compounds

Two types of anti-inflammatory interventions, i.e., omega-3 fatty acid with aspirin and biosynthetic RvD1, were administrated before induction of lung injury. RvD1 (500 ng; Cayman Chemical, Ann Arbor, MI, USA) was injected intravenously (IV) 30 m before the start of the MV injury. Seven days prior to the experiment, mice belonging to the omega-3 fatty acid with aspirin group received daily administrations of 500 µg/g body weight omega-3 acid ethyl esters (Omacor; Kuhnil Pharm., Seoul, Korea) and 15 mg/kg body weight aspirin (Bayer Korea, Seoul, Korea). We calculated the study doses to produce a human (80 kg) equivalent dose of 4 g/day of omega-3 and 120 mg/day of aspirin. A conversion formula of human doses to animal doses based on body surface area suggests that mice should receive approximately ten times the recommended human dosage per kilogram body weight [10,11]. These drugs were mixed into the drinking water, and their concentrations were calculated according to the daily water intake of each mouse.

### 2.4. In Vivo Imaging to Evaluate NF-κB-Luciferase Assay

We used an in vivo imaging system (IVIS Lumina III; PerkinElmer Inc., Waltham, MA, USA), which has a chamber equipped with an electron-multiplying charge-coupled device camera, to monitor pulmonary NF-κB activation. In a previous study, bioluminescence in vivo imaging showed adequate sensitivity for the assessment of the NF-κB activation following an intra tracheal lipopolysaccharide (LPS) challenge [12]. Murine lung tissue was transfected with an NF-κB-Luc using an in vivo-jetPEI (Polyplus Transfection, San Diego, CA, USA) as the carrier. Fifty µg of NF-κB-Luc and 7 µL of jetPEI were each diluted in 200 mL 5% glucose solution. The two solutions were mixed and incubated for 15 min at room temperature. C57BL/6 mice were injected with 400 µL of this mixture through the tail vein. For the measurement of signal, luciferin (Caliper Life Sciences, Hopkinton, MA, USA) was injected before each measurement of NF-κB signal activity. Mice were anesthetized with isoflurane (2–5% induction, 0.25–4% maintenance) and imaged for 10 min after intraperitoneal injection of 150 mg/kg luciferin. Three measurements of the NF-κB activation were conducted for each mouse. The first was measured to confirm an increase in signal intensity the following day from NF-κB-Luc transfection; the second measurement was performed to verify the baseline level of signal intensity just before the induction ALI; the third was conducted 4 h after the completion of ALI. All data were analyzed using the Living Image software, which is integrated into the IVIS. The regions of interest (ROI) were drawn over the chest area. Quantification of the bioluminescence signal inside this ROI was reported as the average photon flux and presented as photons per second per centimeter squared per steradian (p/s/cm^2^/sr).

### 2.5. Bronchoalveolar Lavage Fluid Analysis and Enzyme-Linked Immunosorbent Assays (ELISAs)

BALF was collected after sacrificing the animals. Bronchoalveolar lavage was performed with 1 mL of cold Hanks’ balanced salt solution injected through the tracheal angiocatheter. After centrifugation of the collected fluid at 500 g for 20 min at 4 °C, the supernatant was stored at −80 °C until further analysis of total protein and inflammatory cytokines was performed. Tumor necrosis factor-α (TNF-α) and interleukin-6 (IL-6) assays were conducted using the appropriate mouse ELISA kit (R&D Systems) as recommended by the manufacturer.

### 2.6. Histopathological Evaluation and Quantitative Estimation of Inflammation Using Machine Learning Software

After the collection of BALF, murine lungs were harvested by surgical resection. The lung tissue was perfusion-fixed with 10% formalin injected via the angiocatheter at a fluid pressure of 25 cm H_2_O for 5 min, embedded in paraffin, and stained with haematoxylin and eosin (H&E). All histological findings were investigated by an expert pathologist who was blinded to the experimental groups. Lung injury scores were quantified according to the published criteria, which was assessed on a scale of 0–2 for each of the following criteria: (i) neutrophils in the alveolar space, (ii) neutrophils in the interstitial space, (iii) number of hyaline membranes, (iv) amount of proteinaceous debris, and (v) extent of alveolar septal thickening. The final injury score was derived from the following calculation: Score = [20*(i) + 14*(ii) + 7*(iii) + 7*(iv) + 2*(v)]/(number of fields * 100) which provided an overall score of between 0 and 1 [13].

Additionally, the H&E pictures were quantified using the spectral unmixing method of the analysis software (InForm 2.3 software; PerkinElmer Inc., Waltham, MA, USA) and segmented into normal, injured, and background (Figure S1). Ten images at 10X magnification were taken per whole-lung section for each mouse using the Mantra system (PerkinElmer Inc., Waltham, MA, USA). The total areas of defined tissue segments, such as normal or injured, were summed from 10 images for each slide. After background exclusion, we compared the relative proportions of injured regions to the total areas. The proportions were automatically calculated for whole scans after preliminary machine learning using the sample images.

### 2.7. Statistical Analyses

Continuous data were presented as the mean and the standard error. The smoothing spline function test (spar = 0.1) was used to investigate the time point of the maximal bioluminescence signal intensity after the induction of lung injury [14]. The Kruskal–Wallis tests and the two-tailed Mann–Whitney U tests were used to compare the mean values of continuous variables. Association between the automatically calculated proportion of injured regions to total areas and histopathological lung injury score was assessed using Pearson’s correlation analysis. Statistical analyses were performed using R version 3.5.1 (R Foundation for Statistical Computing, Vienna, Austria) and IBM SPSS Statistics for Windows, version 23.0 (IBM Corp., Armonk, NY, USA). Differences were considered statistically significant with values of *p* < 0.05.

## 3. Results

### 3.1. Setting of VILI Model

In serial in vivo imaging experiments, MV injury increased the bioluminescence signal intensity in NF-κB-luc-transfected mice (*n* = 7). Quantification of the signal from the lung demonstrated that NF-κB expression reached its peak 4 h after MV injury (Figure S2).

### 3.2. Effect of RvD1 Pretreatment and Aspirin and Omega-3 Fatty Acid Pretreatment on ALI

#### 3.2.1. In Vivo Imaging of NF-κB Activation

**Figure 1:**
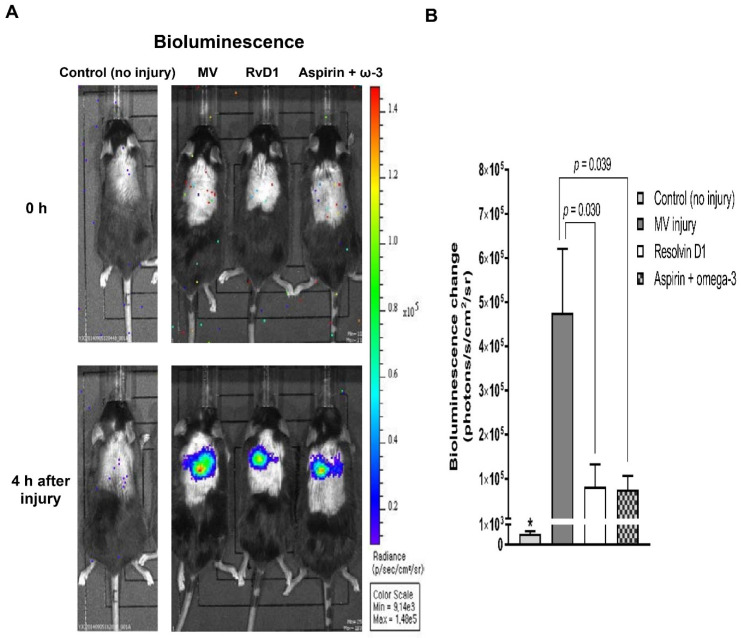
The effect of pretreatment group on NF-κB induction in vivo. In NF-κB-luciferase reporter transfected mice, the quantification of bioluminescence signal from the lungs was conducted at 4 h after MV injury. The number of mice in control, MV injury, resolvin D1 pretreated, and aspirin and omega-3 pretreated groups were 3, 9, 7, and 7, respectively. Data are presented as mean ± standard error of the mean. * *p* < 0.05 for value between control group and injury only group. (**A**) Bioluminescence images. (**B**) Lung signal intensity change. NF-κB, nuclear factor kappa-light-chain-enhancer of activated B cells; MV, mechanical ventilation; RvD1, resolvin D1.

Mice pretreated with aspirin and omega-3 fatty acid showed a significantly minimal change of signal activities (p/s/cm^2^/sr) 4 h after MV injury than those in the injury only group (7.5 × 10^4^ ± 3.1 × 10^4^ vs. 4.7 × 10^5^ ± 1.4 × 10^5^, *p* = 0.039). RvD1 pretreated group also showed significantly decreased signal activity compared to the injury only group (8.2 × 10^4^ ± 5.0 × 10^4^ vs. 4.7 × 10^5^ ± 1.4 × 10^5^, *p* = 0.030) (Figure 1). The signal activity change for the control group of 549.7 ± 125.1 p/s/cm^2^/sr was significantly reduced than that for either the injury only group (*p* = 0.009) or the pretreated groups (*p* = 0.017).

#### 3.2.2. Total Protein and Pro-Inflammatory Cytokines Analysis in the Bronchoalveolar Lavage Fluid

**Figure 2:**
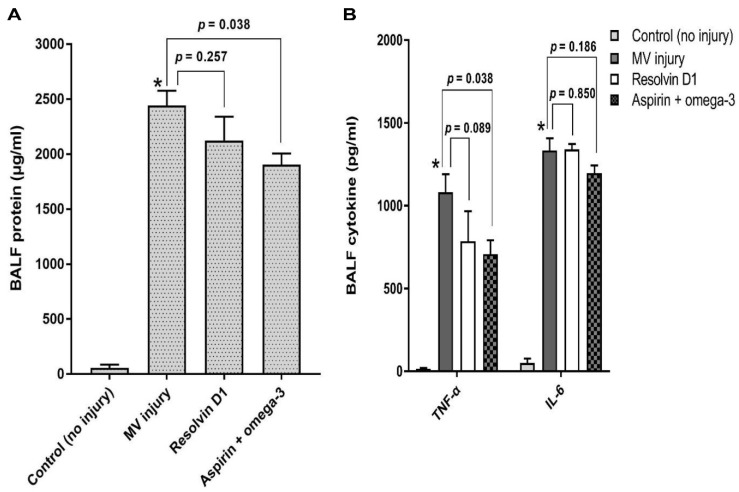
Total protein and cytokines analysis in bronchial alveolar lavage fluid for the effect of pretreatment. TNF-α and IL-6 levels were measured in the BALF after MV injury. The number of mice in the control (no injury), only injury, resolvin D1 pretreated, and aspirin and omega-3 pretreated groups were 3, 7, 4, and 4, respectively. Data are presented as mean ± standard error of the mean. * *p* < 0.05 for value between control group and injury only group. (**A**) BALF protein analysis. (**B**) BALF cytokine analysis. TNF-α, tumor necrosis factor-α; IL-6, interleukin-6; BALF, bronchoalveolar lavage fluid; MV, mechanical ventilation.

Total protein in the BALF of the aspirin and omega-3 fatty acid group was significantly lower than that of the injury only group (*p* = 0.038) (Figure 2A). The concentration of TNF-α was significantly lower in the BALF of aspirin and omega-3 fatty acid treated group than that of the injury only group (*p* = 0.038). However, there was no statistically significant difference in IL-6 levels between the two groups (*p* = 0.186). Moreover, the levels of TNF-α (*p* = 0.089) and IL-6 (*p* = 0.850) in the RvD1 pretreated group were similar to those in the injury only group (Figure 2B).

#### 3.2.3. Histopathological Examination

**Figure 3:**
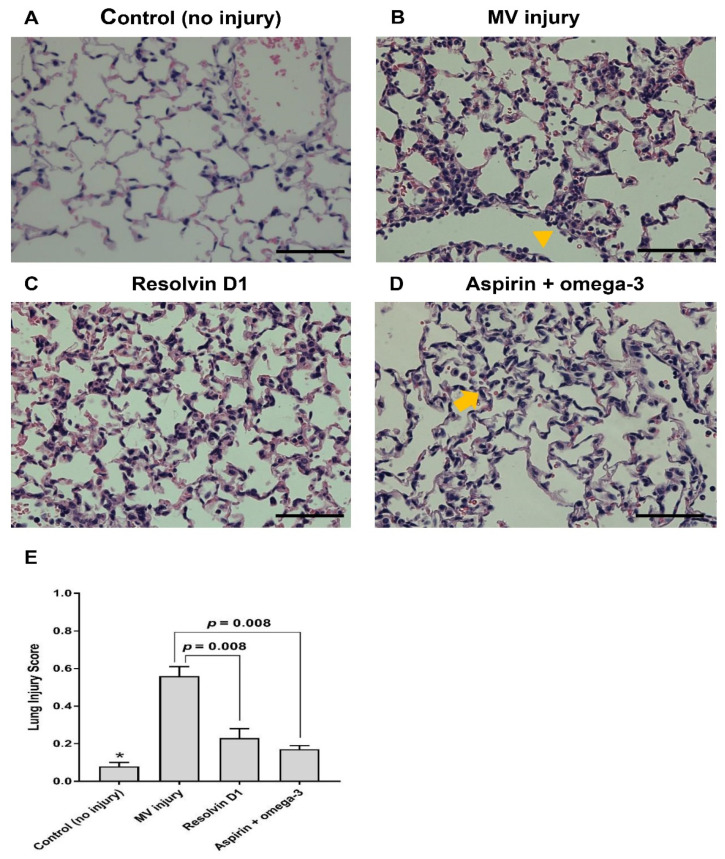
Representative photomicrographs of lung tissue and lung injury scores after mechanical ventilation injury. Hematoxylin and eosin staining were performed (400×, scale bar = 100 µm). (**A**) Control group. (**B**) Injury only group. Active inflammatory lesion including the neutrophilic infiltration was seen in the alveolar ducts (arrow). (**C**) Resolvin D1 pretreated group. (**D**) Enteral aspirin and omega-3 pretreated group. Minimal infiltration of neutrophils was seen in the alveolar walls (arrowhead). (**E**) Lung injury score. The number of mice in the control (no injury), only injury, resolvin D1 pretreated, and aspirin and omega-3 pretreated groups were 3, 7, 4, and 4, respectively. Data are presented as mean ± standard error of the mean. * *p* < 0.05 for value between control group and injury only group.

Histopathological examination revealed an attenuated inflammatory reaction in mice pretreated with RvD1 or aspirin and omega-3 fatty acid compared with the injury only group. RvD1 group and the aspirin and omega-3 fatty acid groups showed significantly decreased lung injury scores than the injury only group (*p* = 0.008) (Figure 3).

#### 3.2.4. A Machine Learning Algorithm for the Interpretation of the Pathological Findings

**Figure 4:**
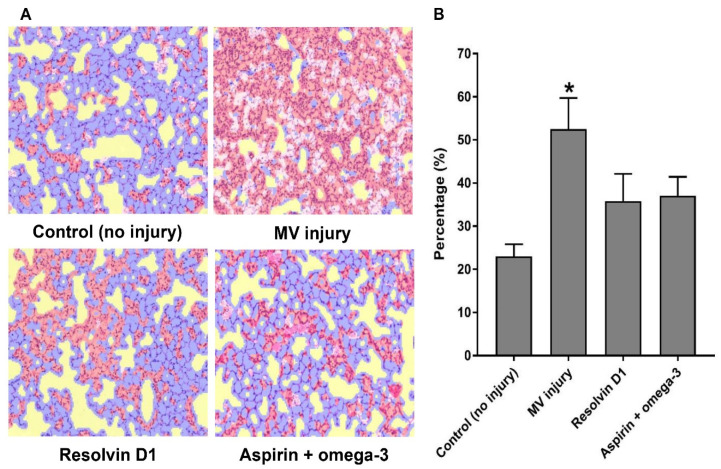
The comparison of proportions of injured regions in lung tissue using automatic detecting software. The total number of mice was 18 (control, 3; only injury, 5; injury after resolvin D1 pretreatment, 4; injury after aspirin and omega-3 fatty acid pretreatment, 4). * *p* < 0.05 for value vs. control group. (**A**) Automatic detecting areas. The automatically scanned total areas of the region of interest in hematoxylin and eosin-stained lung tissue (red color, injured area; blue color, normal area). (**B**) Mean percentage of injury/total area. MV, mechanical ventilation.

After training the machine learning algorithm according to the software instructions, the lung tissue samples were investigated using 160 images from 16 mice. The summed area, including injury, normal tissue, background, and red blood cells, were calculated for each experimental category (no injury, *n* = 3; only injury, *n* = 5; RvD1 pretreated, *n* = 4; aspirin and omega-3 fatty acid-treated, *n* = 4). Mean percentages in the ratio of injury/total area was statistically significant and positively associated with lung injury score (correlation coefficient = 0.876, *p* < 0.01). Injury only group showed increased mean percentage in the ratio of injury/total area than the control group (*p* = 0.025). Though no statistical significance was observed among the injury only group and both pretreatment groups, the pretreatment groups (RvD1 pretreated, 35.8 ± 6.3%; aspirin and omega-3 fatty acid pretreated, 37.1 ± 4.4%) showed relatively lower mean percentages compared to the injury only group (52.5 ± 7.2%) (Figure 4).

## 4. Discussion

In summary, MV injury caused significant pulmonary inflammation as determined by the increased expression of the NF-κB pathway (peaking at 4 h after the induction of injury). In comparison with the injury only group, the pretreatment group with RvD1 or aspirin with omega-3 fatty acid showed reductions in the bioluminescence signal intensity after injury. Moreover, the amount of total protein and TNF-α levels in the BALF of mice pretreated with aspirin and omega-3 fatty acid was significantly lower than the injury only group. Histopathological examination of lungs from the pretreatment groups demonstrated attenuated lung injury. In addition, calculated percentages of the injured area using a machine learning algorithm showed a similar trend with that of bioluminescence signal intensities.

ARDS is a representative example of hyperactive acute inflammatory response to noxious stimuli or infection [15]. Resolvins, omega-3 fatty acid-derived mediators, may provide beneficial effects on attenuating uncontrolled inflammation via several mechanisms, including suppression of neutrophil activation, increasing phagocytosis by macrophages, and stabilization of the cellular structure. According to several studies, RvD1 could decrease the levels of macrophage-derived pro-inflammatory cytokines, regulate phagocytosis in human macrophages [16,17], and have cytoprotective effects in the resolution phase. RvD1 administration reduced aberrant neutrophil-mucosa interactions by preventing the accumulation of activated neutrophils [18].

Based on these results, enteral supplementation with omega-3 fatty acid was considered beneficial in modulating the inflammatory processes. Pontes-Arruda et al. reported that, in patients with sepsis, enteral omega-3 and gamma-linolenic acid resulted in a significant reduction in the risk of mortality as well as in the duration of the MV and the intensive care unit stay [6,19]. However, in the ARDSNet OMEGA trial, supplementation with omega-3 fatty acid, gamma-linolenic acid, and antioxidants, twice a day, did not improve the number of ventilator-free days, mortality, or physiological lung parameters such as plateau pressure, minute ventilation, and PaO_2_/FiO_2_ ratio in patients with ALI. Unlike previous positive studies using the relatively high-fat control supplements containing predominantly omega-6 and omega-9 fatty acids that could be metabolized into inflammatory prostaglandins and series-4 leukotrienes, the control supplement in OMEGA trial contained mainly carbohydrates. This was suggested as one of the reasons for the negative results [20].

We hypothesized that simultaneous pretreatment with enteral aspirin and omega-3 fatty acid could be a plausible option to reduce the magnitude of VILI, since this concurrent enteral supplementation could also produce additional aspirin-triggered (AT) resolvins. AT resolvins are the epimers of resolvins. While resolvin biosynthesis involves oxygenation by 15-lipoxygenase, AT resolvins are synthesized by the aspirin-acetylated cyclooxygenase-2 enzyme. AT resolvins are known to have equipotent anti-inflammatory and pro-resolving properties and are more resistant to rapid inactivation by eicosanoid oxidoreductases than resolvins [21,22]. In the present study, enteral aspirin and omega-3 fatty acid supplement reduced the degree of VILI. Although we could not measure the level of resolvins directly, including AT form, existing data from previous studies suggest that intravenous or intraperitoneal administration of resolvins or AT resolvins provides potent mucosal protection and promotes catabasis in murine models with distinct ALIs, such as hyperoxic-, immune complex-, or acid-induced lung injury [23,24,25]. In a viral and bacterial co-infection model of pneumonia, intravenous administration of AT-RvD1 curbed the severity of pneumonia by limiting leukocyte chemotaxis to the lungs [26]. Although there have been reports of significant benefits to simultaneous enteral administration of omega-3 fatty acid and aspirin in reducing the plasma levels of inflammatory cytokine and angiogenesis factors, and atherosclerosis [27,28], to our best knowledge, no previous study has examined the effects of combined enteral omega-3 fatty acid and aspirin pretreatment in a mouse model with VILI. In the absence of definitive prevention or treatment measures for lung injury or ARDS in patients on MV, our findings suggest a feasible preventive option that can be further examined in clinical studies. 

IL-6 levels in our study did not change in both pretreatment groups, a result that may be due to differences in the time of the shift in cytokines due to the injury. Blackwell et al. showed that after the application of intraperitoneal LPS, TNF-α rapidly declined within 4 h, and IL-6 showed later onset and decreased by 6 h [29]. Based on our findings, the in vivo imaging method that enabled visualization of changes at the gene level may have advantages over traditional methods regarding convenience and sensitivity. For instance, in a relatively short duration (4 h) in the acute lung injury model, in vivo imaging methods seemed to be preferable to traditional methods, such as measuring wet/dry ratios or levels of cytokines in alveolar lavage fluid and evaluating optical microscopic pathology to calculate the extent of inflammatory responses [12]. Additional studies are needed to further examine the usefulness of the in vivo imaging system in acute lung injury.

In this study, we attempted to quantify the magnitude of inflammation using the automatic software-based machine learning algorithm for interpreting histological findings. As a result, the pretreatment groups showed a reduced percentage of inflammation area than the injury only group, although no statistical significance was observed. If more technological advances and validation data sets are made in the future, automatic detecting software may be a useful tool to increase both the amount and objectivity of data acquisition in histologic tissue injury grade, and to reduce the workload of pathologists on labor-intensive tasks, especially multiplexed immunohistochemistry (chromogenic and fluorescence) assays.

Our study has several limitations. Firstly, we could not measure the plasma concentrations of omega-3 fatty acid derivatives, including AT forms. Additional comparative studies based on in vivo levels of lipid mediators can provide information on the comparative advantage of the synergistic effect of simultaneous supplementation. Secondly, since we did not use male mice, the generalizability of our results was limited. Thirdly, to investigate the effects of simultaneous pretreatment of aspirin and omega-3 fatty acid on VILI more precisely, and to confirm the individual impact of each drug, comparisons to other groups such as the aspirin only arm or omega-3 only arm is needed. Lastly, additional data from blood gas analysis may also provide more accurate information on the effect of hyperventilation or barotrauma caused by MV. Some mice died due to pneumothorax and therefore were excluded from our study. Their exclusion can explain the differences in group size among experimental groups. Nevertheless, the statistical power of this study was calculated to be 0.86, which allows us to accept that the experiment data are reliable. Further studies should conduct higher levels of monitoring to detect the unstable hemodynamic state or critical events.

## 5. Conclusions

Simultaneous pretreatment with enteral supplementation of aspirin and omega-3 fatty acid reduced lung injury in a murine VILI model and could be a potential prevention method for VILI. Future studies focusing on the preventive effect of this treatment after the start of mechanical ventilation are needed.

## Supplementary Materials

The following are available online at https://www.mdpi.com/article/10.3390/nu13072258/s1, Figure S1: The quantitative inflammatory information of lung tissue (hematoxylin & eosin staining), Figure S2: In vivo NF-κB activation in response to MV injury.
