# Supplementary material for: Simultaneous Pretreatment of Aspirin and Omega-3 Fatty Acid Attenuates Nuclear Factor-κB Activation in a Murine Model with Ventilator-Induced Lung Injury
Source: Nutrients. 2021 Jun 30;13(7):2258. doi: 10.3390/nu13072258 (PMC8308446; doi:10.3390/nu13072258)
Supplement: Supplementary file 1 [file nutrients-13-02258-s001.zip › nutrients-1263935-supplementary.pdf]

Title: Simultaneous pretreatment of aspirin and omega-3 fatty acid attenuates nuclear factor-κB activation in a murine with ventilator-induced lung injury

Won Gun Kwack, Yoon Je Lee, Eunyoung Eo, Jin-Haeng Chung, Jae Ho Lee, and Young-Jae Cho#

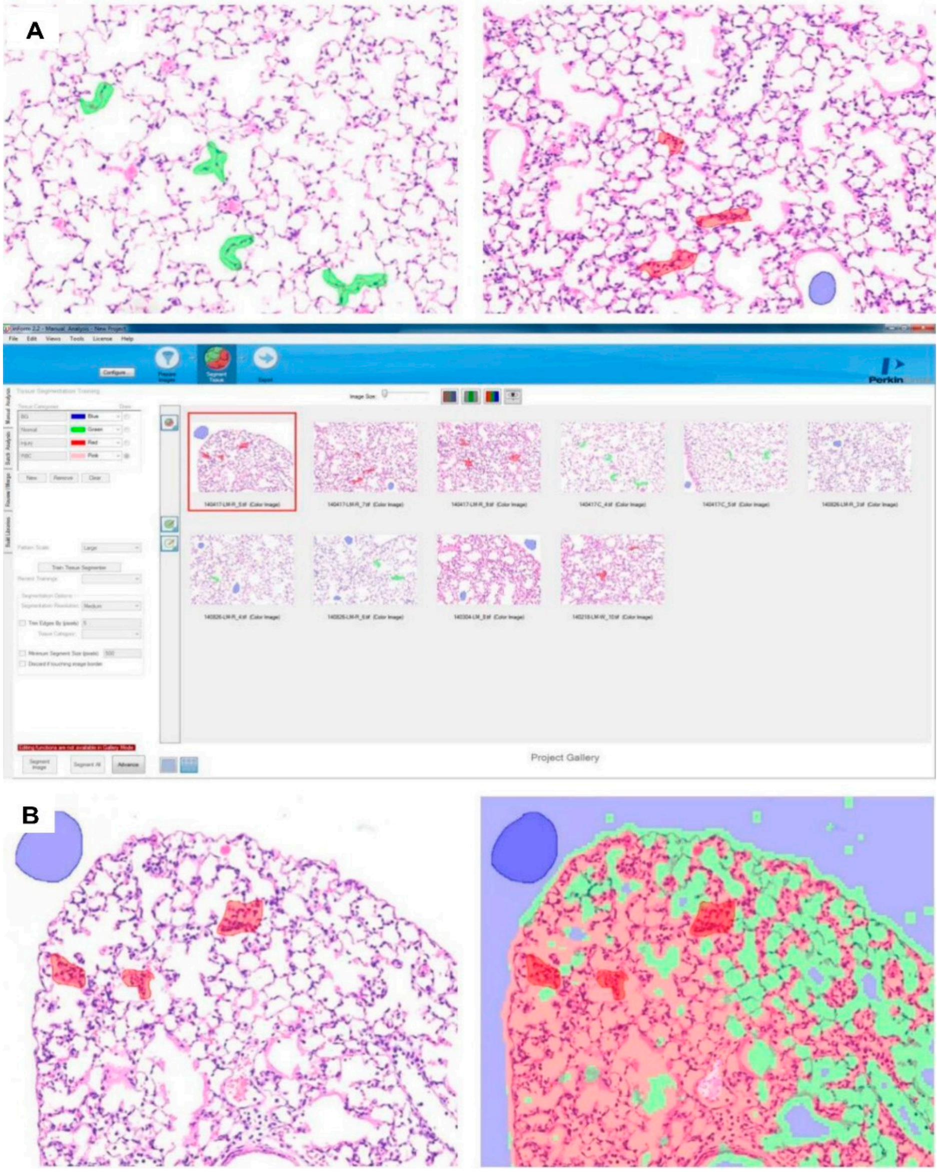

Figure S1. The quantitative inflammatory information of lung tissue (hematoxylin & eosin staining). These color mapping was arbitrary for demonstration and didn't match with real experimental data. (a) Automatic slide scanning. Ten images with x10 magnification per slide were scanning. (b) Automatic detecting area. Segmentation were proceeded dividing normal (green), injured (red), or background (blue).

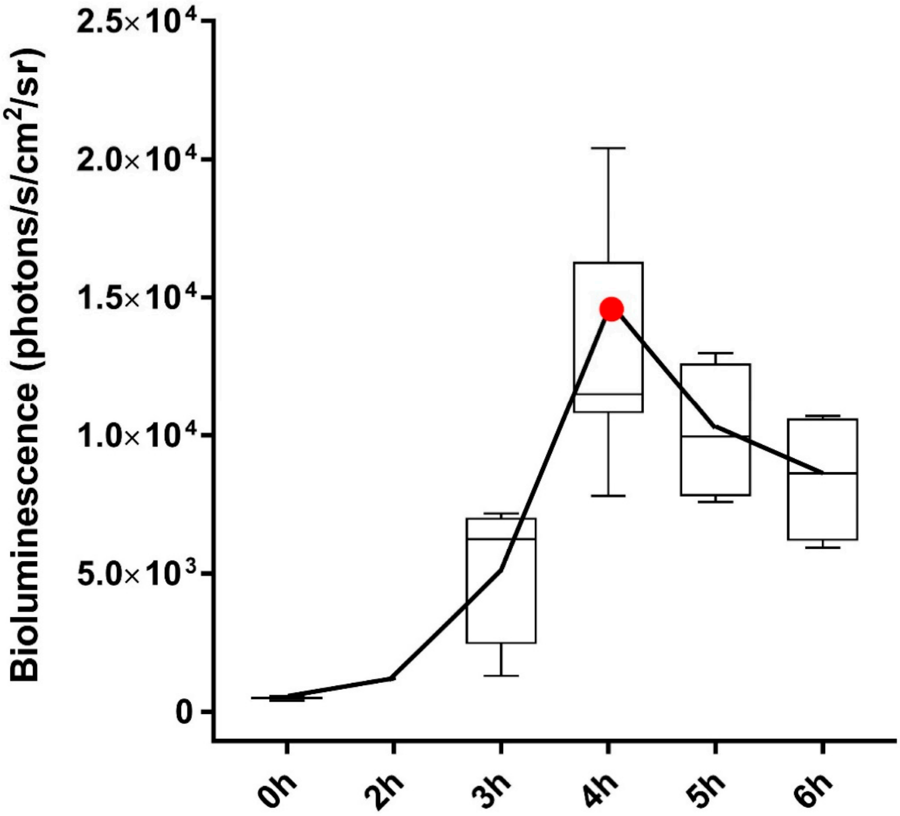

Figure S2. ***In vivo*** **NF-κB activation in response to MV injury.** In NF-κB-luciferase reporter transfected mice (n = 7), the quantification of bioluminescence signal from the lungs were conducted at 0, 3, 4, 5, and 6 hours after MV (4 h; tidal volume, 30 ml/kg; respiratory rate, 120 breaths/min) injury. Red dot is the inflection point. NF-κB, nuclear factor kappa-light-chain-enhancer of activated B cells; MV, mechanical ventilation.
